# PRKAG2 Cardiac Syndrome: A Comprehensive Review of Clinical Manifestations, Pathophysiological Mechanisms, and Precision Medicine Frontiers

**DOI:** 10.1007/s12265-026-10851-y

**Published:** 2026-11-05

**Authors:** Yzabella Alves Campos Nogueira, Mariana Barreto Martins, André Lacerda de Abreu Oliveira, Arnoldo Rocha Façanha, Eduardo Back Sternick, Glauber Monteiro Dias

**Affiliations:** 1 Laboratório de Biologia Celular e Tecidual, Universidade Estadual do Norte Fluminense Darcy Ribeiro, Campos dos Goytacazes — RJ, Av. Alberto Lamego, 2000 — Parque Califórnia, Campos dos Goytacazes, Rio de Janeiro, 28013-602 Brazil; 2 Laboratório de Clínica e Cirurgia Animal, Universidade Estadual do Norte Fluminense Darcy Ribeiro, Campos dos Goytacazes — RJ, Rio de Janeiro, Brazil; 3 Hospital Materdei, Belo Horizonte, MG Brazil

**Keywords:** PRKAG2 cardiac syndrome, AMP-activated protein kinase, Glycogen-storage cardiomyopathy, Fasciculoventricular pathways, Ventricular preexcitation, Precision medicine

## Abstract

PRKAG2 cardiac syndrome (PCS) is a metabolic storage cardiomyopathy caused by pathogenic variants in the PRKAG2 gene encoding the γ2-subunit of AMP-activated protein kinase (AMPK). It is defined by left ventricular hypertrophy, ventricular preexcitation and progressive conduction system disease accompanied by early-onset atrial arrhythmias. This review systematically evaluates PCS, outlining its clinical manifestations, genotype-phenotype spectrum, and diagnostic clues that distinguish it from sarcomeric hypertrophy. It explores underlying pathophysiological mechanisms, detailing how chronic AMPK activation drives glycogen accumulation via allosteric G6P activation of glycogen synthase, following a biphasic temporal model from early GLUT4-mediated glucose flooding to late metabolic feedback suppression. It further examines how Akt–mTOR hyperactivation fosters hypertrophy and how activated fasciculoventricular pathways mediate preexcitation. Finally, it reviews current supportive clinical management and highlights emerging precision medicine frontiers—including RNA-based silencing, pharmacological pathway inhibitors, and CRISPR-Cas9 gene editing—that aim to shift PCS care toward targeted disease modification.

**Supplementary Information:**

The online version contains supplementary material available at https://doi.org/10.1007/s12265-026-10851-y.

## Introduction

PRKAG2 cardiac syndrome (PCS) represents a unique and complex clinical entity within the spectrum of cardiomyopathies, characterized by a triad of left ventricular hypertrophy (LVH), ventricular preexcitation (VPE), and advanced conduction disorders, accompanied by a high incidence of atrial arrhythmias [1, 2]. In contrast to classic hypertrophic cardiomyopathy (HCM), which is primarily caused by variants in sarcomeric genes, PCS stems from dysregulated energy metabolism and is histologically defined by the pathological accumulation of intracellular glycogen [2, 3]. Dysfunctional adenosine monophosphate-activated protein kinase (AMPK), resulting from pathogenic variants in the regulatory $\:{\gamma\:}_{2}$-subunit ($\:{\gamma\:}_{2}$-AMPK) encoded by the *PRKAG2* gene, plays a central role in the syndrome by disrupting cellular energy homeostasis [4].

Recent studies have significantly expanded our understanding of the pathogenesis of PCS [4, 5]. Data from animal models and clinical studies point to a multifactorial pathophysiology in which glycogen accumulation does not act in isolation to induce myocardial hypertrophy; rather, it operates in synergy with the dysregulated activation of growth pathways such as the Akt–mTOR axis [6]. Furthermore, the marked phenotypic variability observed among carriers of different *PRKAG2* variants highlights the necessity for a personalized approach to both diagnosis and patient management.

These structural cardiac disorders are distributed globally [7]. Although the prevalence of PCS among patients initially diagnosed with HCM is less than 1% [8], the expanding global availability of genetic testing—the current gold standard for diagnosis—is expected to increase disease detection rates. The high incidence of severe complications and sudden cardiac death (SCD) associated with complete atrioventricular (AV) block and atrial fibrillation (AF)[3] underscores the urgent need for deeper investigation into the clinical implications of *PRKAG2* gene variants.

This review aims to provide a comprehensive overview of the clinical, molecular, and pathophysiological aspects of PCS based on studies in humans harboring *PRKAG2* variants and animal models expressing mutant $\:{\gamma\:}_{2}$-AMPK, while exploring current management practices and future therapeutic strategies rooted in precision medicine.

### Clinical Manifestations and Diagnostic Approaches

To date, more than twenty variants in the *PRKAG2* gene have been linked to PCS or other cardiovascular phenotypes. While each variant is associated with a distinct array of structural and electrophysiological alterations, the core clinical triad of PCS — LVH, intracellular glycogen accumulation in cardiomyocytes, VPE, and conduction disturbances — is present in most affected individuals. Sinus bradycardia (resting heart rate * <60* beats per minute) and atrial tachyarrhythmias (such as AF or atrial flutter) frequently represent the initial clinical presentation [3–5, 8, 9]. Patients may also present with symptoms including dyspnea, fatigue, chest pain, and syncope. Notably, SCD has been reported in previously asymptomatic individuals. Additional clinical red flags — including disproportionately high QRS voltages on electrocardiography (ECG), early-onset isolated atrial flutter, and atypical infantile dilated phenotypes — have been identified to streamline clinical screening (Table 1) [4, 5,10–14]

**Table 1:** Clinical Red Flags and Diagnostic Clues for the Differential Diagnosis of PRKAG2 Cardiac Syndrome (PCS)

Clinical Category	Classic Features and Emerging “Red Flags”	Diagnostic Significance / Differentiation from Sarcomeric HCM	Key References
Electrocardiographic Markers	Ventricular preexcitation with short PR interval	Highly prevalent in PCS (especially Arg302Gln); rare in sarcomeric HCM. Mediated by decremental FVPs rather than classical accessory pathways.	[11,12,19]
	Disproportionately high QRS voltages	Reflects intracellular glycogen storage and myocyte enlargement, often exceeding QRS voltage expectations for wall thickness.	[3, 8]
Conduction System Disease	Early-onset progressive AV blocks and sinus bradycardia	High rate of bundle branch blocks and complete AV block requiring PPM implantation at a young age.	[8, 11–13]
Arrhythmia Burden	Early-onset Atrial Flutter and Atrial Fibrillation	Supraventricular arrhythmias are frequently the initial symptom, presenting significantly earlier in life than in standard HCM.	[8,11,12,﻿^17^,^19^]
Multimodal Imaging	Absence of significant myocyte disarray and interstitial fibrosis	Cardiac MRI demonstrates minimal or absent LGE in early/mid stages, contrasting with classical sarcomeric HCM.	[10, 18]
	Preserved global longitudinal strain on Echo	Preserved systolic function compared with sarcomeric HCM	[17]
Atypical Morphological Phenotypes	Infantile DCM / Rapid Heart Failure	Specific non-CBS variants (e.g., Thr142Ile) can present in infancy as aggressive DCM, diverging from adult hypertrophic phenotypes.	[14]

*AV *Atrioventricular, *DCM *Dilated cardiomoathy, *FVPs *Fasciculoventricular pathways, *LGE *Late gadolinium enhancement, *LVH *Left ventricular hypertrophy, *HMC *Hypertrophic cardiomyopathy, *MRI *Magnetic ressonance Imaging, *PPM *Permanent pacemaker

The molecular and cellular abnormalities underlying PCS typically manifest clinically during adolescence or early adulthood. Within this clinical framework, assessing familial inheritance patterns is critical, particularly when family history reveals early-onset permanent pacemaker (PPM) implantation, unexplained cardiomyopathy, or premature SCD.

In patients with PCS, echocardiographically detected LVH is commonly misdiagnosed as classical HCM [10]. However, unlike sarcomeric variants — which are typically associated with interstitial fibrosis, myofibrillar disarray, and myocyte disruption — PCS lacks these classic histopathological features, although minimal myocardial fibrosis has been reported in select cases. Instead, PCS is characterized by glycogen-laden vacuoles within preserved, organized cardiomyocytes [2, 16]. Furthermore, patients with PCS present with preserved global longitudinal strain (GLS), indicating normal left ventricular systolic function, whereas patients with sarcomeric HCM typically exhibit impaired GLS [17].Cardiac magnetic resonance imaging (MRI) serves as a pivotal tool for differentiating PCS from sarcomeric HCM. This distinction relies heavily on the assessment of late gadolinium enhancement (LGE), a marker of myocardial fibrosis that is characteristically minimal or absent in the early-to-mid stages of storage-based cardiomyopathies compared to sarcomeric phenocopies (Table 1) [18]. Clinical suspicion of a non-sarcomeric HCM phenocopy is further raised by a high incidence of electrophysiological abnormalities that are uncommon in classical HCM, such as VPE (frequently manifesting as Wolff-Parkinson-White [WPW] pattern) and progressive cardiac conduction system disease. PCS exhibits a progressive clinical course associated with higher complication rates compared to sarcomeric HCM [3]. Conduction system disease represents the most frequent outcome, with approximately 32% to 43% of patients requiring PPM implantation due to advanced atrioventricular blocks or sinus node dysfunction [9, 19]. Atrial fibrillation and atrial flutter are common and characteristically occur early in life, often before 40 years of age. Notably, atrial flutter has been identified as a strong predictor of subsequent pacemaker dependency [12]. Sudden cardiac death occurs in roughly 8.7% to 13% of affected individuals. Progression to systolic heart failure occurs in approximately 12% to 15% of patients, with cardiac transplantation rates ranging between 4% and 6% [3, 9, 11]

### Genotype-Phenotype Association

The clinical presentation of PCS is remarkably heterogeneous, ranging from completely asymptomatic individuals to cases of sudden cardiac death in adolescents and young adults. The Arg302Gln variant is the most prevalent mutation worldwide, accounting for approximately 57% of reported cases. It is strongly linked to high rates of early pacemaker dependency and VPE [9, 11]. The profound impact of the Arg302Gln variant on the conduction system is well documented across diverse cohorts, highlighting its propensity for severe electrophysiological disruption. Familial studies demonstrate that Arg302Gln carriers presenting with hypertrophy exhibit a broad spectrum of electrocardiographic abnormalities, including sinus bradycardia, supraventricular tachycardia, bundle branch blocks, preexcitation, and variable degrees of AV block [2, 11]. This extensive impairment was corroborated by a comparative observational study in patients with atrial flutter: individuals harboring the Arg302Gln variant developed atrial flutter significantly earlier in life and exhibited a markedly higher incidence of preexcitation, right bundle branch block, and progressive AV conduction failure compared to non-PCS controls. Crucially, complete AV block was exclusively observed in the variant cohort [12].

The second most common mutation is Asn488Ile, representing roughly 21% of cases [9, 11]. The Lys485Glu variant and more recently identified mutations — including Arg302Pro, Leu341Ser, and His401Asp — have been characterized as highly pathogenic, exhibiting complete clinical penetrance for ventricular hypertrophy (asymmetric septal, concentric, or apical), sinus bradycardia, progressive conduction failure, and elevated risk of heart failure and SCD [11].

Other variants are ultra-rare and geographically restricted. For example, the p.Lys290Ile and p.Lys291Ile variants identified in a large Brazilian family cohort were linked to a guarded clinical prognosis and notable extracardiac manifestations [4]. Furthermore, the novel Brazilian His401Gln variant displays a highly aggressive cardiac phenotype. In affected carriers, LVH manifests during early childhood as severe, predominantly septal thickening. All carriers exhibit fasciculoventricular preexcitation, progressive symptomatic sinus bradycardia requiring early pacing, and early-onset atrial arrhythmias (atrial fibrillation from age 11 and tachycardia). This variant is further distinguished by a high incidence of SCD in young adults in their 20s [13].

Complementing the phenotypic spectrum, a case report described a 9-month-old proband presenting with severe dilated cardiomyopathy (DCM) and therapy-refractory heart failure. Exome sequencing identified a *de novo* c.425 C > T (Thr142Ile) variant located in the non-cystathionine β-synthase (CBS) region of *PRKAG2*, illustrating an atypical morphological manifestation expanding the known spectrum of the disease [14]. Table 2 summarizes the clinical findings and characteristics associated with the most common and regional variants.

**Table 2:** PRKAG2 variants associated with a cardiovascular phenotype

Variant (cDNA)	Protein Effect	Domain Location	Clinical Phenotype / Key Features
c.905G > A	p.Arg302Gln	CBS1	Severe LVH, VPE -FVP, early AV block, atrial flutter/AF, high pacemaker rate
c.1463 A > T	p.Asn488Ile	CBS4	Concentric LVH, VPE, conduction disease, progressive heart failure
c.1453 A > G	p.Lys485Glu	CBS4	Severe early-onset LVH, 100% penetrance for bradycardia and AV block
c.904 C > C	p.Arg302Pro	CBS1	Marked LVH, conduction failure, complete clinical penetrance
c.1022T > C	p.Leu341Ser	CBS2	Asymmetric/concentric LVH, early conduction disease, high SCD risk
c.1201 C > T	p.His401Asp	CBS3	Severe LVH, VPE, progressive heart failure
c.1203 C > G	p.His401Gln	CBS3	Aggressive Brazilian phenotype; childhood severe LVH, 100% FVP preexcitation, early AF (age 11), high young adult SCD risk
c.869 A > T	p.Lys290Ile	CBS1	Regional Brazilian variant; severe cardiomyopathy, high arrhythmia burden, extracardiac signs
c.872 A > T	p.Lys291Ile	CBS1	Regional Brazilian variant; guarded prognosis, conduction disease, systemic manifestations
c.425 C > T	p.Thr142Ile	Non-CBS	Atypical infantile presentation; severe DCM and refractory heart failure

*AF *Atrial fibrillation, *AV *Atrioventricular, *CBS *cystathionine β-synthase*, DCM *Dilated cardiomoathy, *FVP *Fasciculoventricular pathway, *LVH *Left ventricular hypertrophy, *SCD *Sudden cardiac death, *VPE* Ventricular preexcitation

Following the recognition of these red flags, comprehensive cardiomyopathy gene panel testing becomes essential to differentiate sarcomeric HCM from non-sarcomeric phenocopies, establish a definitive molecular diagnosis, guide clinical risk stratification, and enable genotype-driven cascade family screening.

### Molecular and Electrophysiological Mechanisms

The majority of pathogenic *PRKAG2* variants cluster within the functional domains of the $\:{\gamma\:}_{2}$-AMPK subunit. Functional assays demonstrate that mutations in these specific regions impair the AMPK heterotrimeric complex, disrupting normal cellular energy homeostasis in cardiac myocytes [11, 20]. Although dysregulated cardiomyocyte metabolism is established as the primary driver of PCS, the specific downstream signaling networks responsible for distinct pathological phenotypes continue to be elucidated.

The heart is one of the most energy-demanding organs, requiring continuous synthesis of adenosine triphosphate (ATP) from metabolic substrates to maintain cardiovascular function. Under physiological conditions, the majority of myocardial ATP originates from mitochondrial fatty acid oxidation, with glucose oxidation providing additional metabolic energy [21, 22]. AMPK functions as a central metabolic sensor, regulating catabolic and anabolic pathways [23]. Activated during cellular energy stress (when ATP falls and AMP rises), AMPK suppresses ATP-consuming synthetic pathways while stimulating ATP-generating catabolic processes to restore energy balance [24].

Beyond regulating lipid and carbohydrate metabolism, AMPK modulates diverse cellular functions including cell growth, autophagy, mitogenic signaling, antioxidant responses, DNA repair, and circadian rhythms [24, 25]. Under acute metabolic stress, AMPK facilitates insulin-independent glucose uptake by driving the translocation of glucose transporter 4 (GLUT4) to the sarcolemma. It simultaneously stimulates glycolysis via phosphorylation of phosphofructokinase 2 (PFK2), which generates fructose-2,6-bisphosphate — a potent allosteric activator of phosphofructokinase 1 (PFK1) [26]. However, functional studies in iPSC-derived cardiomyocytes harboring *PRKAG2* mutations (Arg302Gln and Asn488Ile) reveal altered substrate utilization characterized by suppressed glycolytic activity, elevated mitochondrial respiration, and increased reactive oxygen species generation [27, 28].

Most PCS-associated mutations impair the CBS domains of $\:{\gamma\:}_{2}$-AMPK, causing chronic AMPK overactivation. Sustained AMPK activation produces paradoxical metabolic consequences. Although AMPK acutely suppresses glycogen synthesis through inhibitory phosphorylation of glycogen synthase (GS), chronic activation promotes marked glycogen accumulation in cardiac and skeletal muscle. Increased glucose influx elevates intracellular glucose-6-phosphate (G6P), a powerful allosteric activator of GS that overrides AMPK-mediated inhibitory phosphorylation [29]. Coupled with suppressed glycolysis, accumulated G6P is preferentially converted to glucose-1-phosphate, driving substrate into glycogen synthesis pathways [27].

Conversely, positron emission tomography (PET) studies using 18 F-fluorodeoxyglucose ($\:{}_{}{}^{18}\mathrm{F}$-FDG) by Gollob and colleagues demonstrated decreased myocardial glucose uptake in adult human carriers and mouse models expressing the Arg302Gln variant, with no response to insulin stimulation [30, 31]. These findings support a biphasic temporal model in the natural history of PCS. In the acute/neonatal phase, constitutive $\:{\gamma\:}_{2}$-AMPK activity causes continuous GLUT4 sarcolemmal translocation, flooding the cardiomyocyte with glucose. In the chronic/adult phase, massive glycogen storage exerts allosteric negative feedback on the AMPK complex, downregulating downstream glucose transport and glycolytic flux to protect the cell from osmotic and structural collapse [32, 33]. Mechanistically, AMPK activity is modulated by the carbohydrate-binding module (CBM) of its $\:\beta\:$-subunit, which interacts with glycogen granules. Glycogen binding to the CBM directly inhibits AMPK activity, illustrating a reciprocal regulatory feedback loop between glycogen stores and AMPK signaling [22].

While initial models proposed that physical glycogen accumulation directly drives myocardial hypertrophy, subsequent experiments — particularly in Asn488Ile transgenic models — demonstrated that pharmacological or genetic inhibition of glycogen storage resolved preexcitation without blunting myocardial hypertrophy. These data indicate that glycogen accumulation alone is insufficient to drive the hypertrophic phenotype observed in PCS, bringing the mechanistic target of rapamycin (mTOR) axis to the forefront as a central mediator of cardiac hypertrophy [6, 34]. In cardiomyocytes, AMPK suppresses mechanistic target of rapamycin complex 1 (mTORC1) to inhibit anabolic protein synthesis and promote autophagy [35]. However, in Asn488Ile mouse models subjected to G6P inhibition, post-natal mTOR activation driven by pathogenic AMPK signaling promoted cardiomyocyte proliferation and adult cell hypertrophy despite reduced glycogen storage. This growth was accompanied by increased protein synthesis and suppression of Forkhead box O (FoXO) transcription factors. Notably, both mTOR inhibition and FoXO re-activation substantially attenuated cardiac hypertrophy [34]. Similarly, Lys475Glu mutant mice displayed enhanced AMPK activity coupled with hyperactivation of the mTOR/p70S6K/4EBP1 and Akt–mTOR–FoXO3a pathways, thereby enhancing protein synthesis and myocardial hypertrophy [6]. Akt (protein kinase B) is a major upstream regulator of cell survival and hypertrophy [36]. Akt1-deficient mice (${\mathrm{Akt1}}^{-/-}$) demonstrate growth impairment [37], whereas transgenic overexpression of active Akt1 induces severe cardiac hypertrophy that is sensitive to rapamycin [38]. Furthermore, in vitro treatment of Arg302Gln mutant cardiomyocytes with metoprolol - a β-adrenergic receptor blocker - suppressed Akt–mTOR phosphorylation and AMPK activity, reducing hypertrophic remodeling and suggesting a potential pathway-directed therapeutic approach [6].

In addition to structural remodeling, AMPK modulates cardiac electrophysiology by regulating ion channels. Activated AMPK alters the expression, trafficking, and gating kinetics of voltage-gated sodium channels ($\:{\mathrm{Na}}_{\mathrm{v}}1.5$), potassium channels ($\:{\mathrm{K}}_{\mathrm{v}}7.1$, $\:{\mathrm{K}}_{\mathrm{v}}11.1$, $\:{\mathrm{K}}_{\mathrm{i}}\mathrm{r}2.1$), and L-type calcium channels ($\:{\mathrm{Ca}}_{\mathrm{v}}1.2$), as well as gap junction connexins [39–42]. Despite previous speculation [43], there is no definitive evidence that pathogenic PRKAG2 variants disrupt physiological ion channel phosphorylation/expression, predisposing to conduction blocks and atrial arrhythmias.

A study with Arg299Gln mouse models suggests that sinoatrial node (SAN) dysfunction in PCS has been attributed to physical glycogen infiltration within specialized pacemaker cells, impairing intrinsic automaticity. Additionally, it was observed a reduced SAN firing rates and decreased funny current ($\:{I}_{f}$) density secondary to down-regulation of Hyperpolarization-Activated Cyclic Nucleotide-Gated Potassium Channel 4 (HCN4) — an essential component for the generation of pacemaker potentials in the SAN [44].

### Hypothesis of Fasciculoventricular Pathways as a Preexcitation Mechanism

VPE occurs when an atrial impulse prematurely activates the ventricular myocardium by bypassing the rate-limiting delay of the atrioventricular (AV) node via an accessory pathway. The most common form, WPW syndrome, results from a congenital muscular connection between the atria and the ventricles. Affecting approximately 1 to 3 per 1,000 individuals, WPW syndrome frequently predisposes patients to rapid tachyarrhythmias [45, 46]. Preexcitation is an established feature of several genetic disorders, including PCS [1, 9, 47], Pompe disease [48], Danon disease [49], Tuberous Sclerosis [50], and specific mitochondrial cytopathies [51, 52]. The incidence of preexcitation in PCS varies by variant, reaching nearly 80% in Arg302Gln carriers [9, 19]. However, invasive electrophysiological studies reveal that preexcitation in PCS is predominantly mediated by decremental fasciculoventricular pathways (FVPs) rather than typical AV accessory pathways [53–55]. Recent anatomical studies indicate that concealed, electrically silent FVPs are ubiquitous in mammalian hearts [56, 57]. Histological analyses by Mahmud et al. identified non-pathological fasciculoventricular connections extending from the left His bundle branch to the ventricular septum in 66.6% of normal human hearts [58]. Sternick et al. reported significantly shorter AV conduction intervals in PCS patients with FVPs compared to non-PCS controls with FVPs [55].

**Fig. 1:**
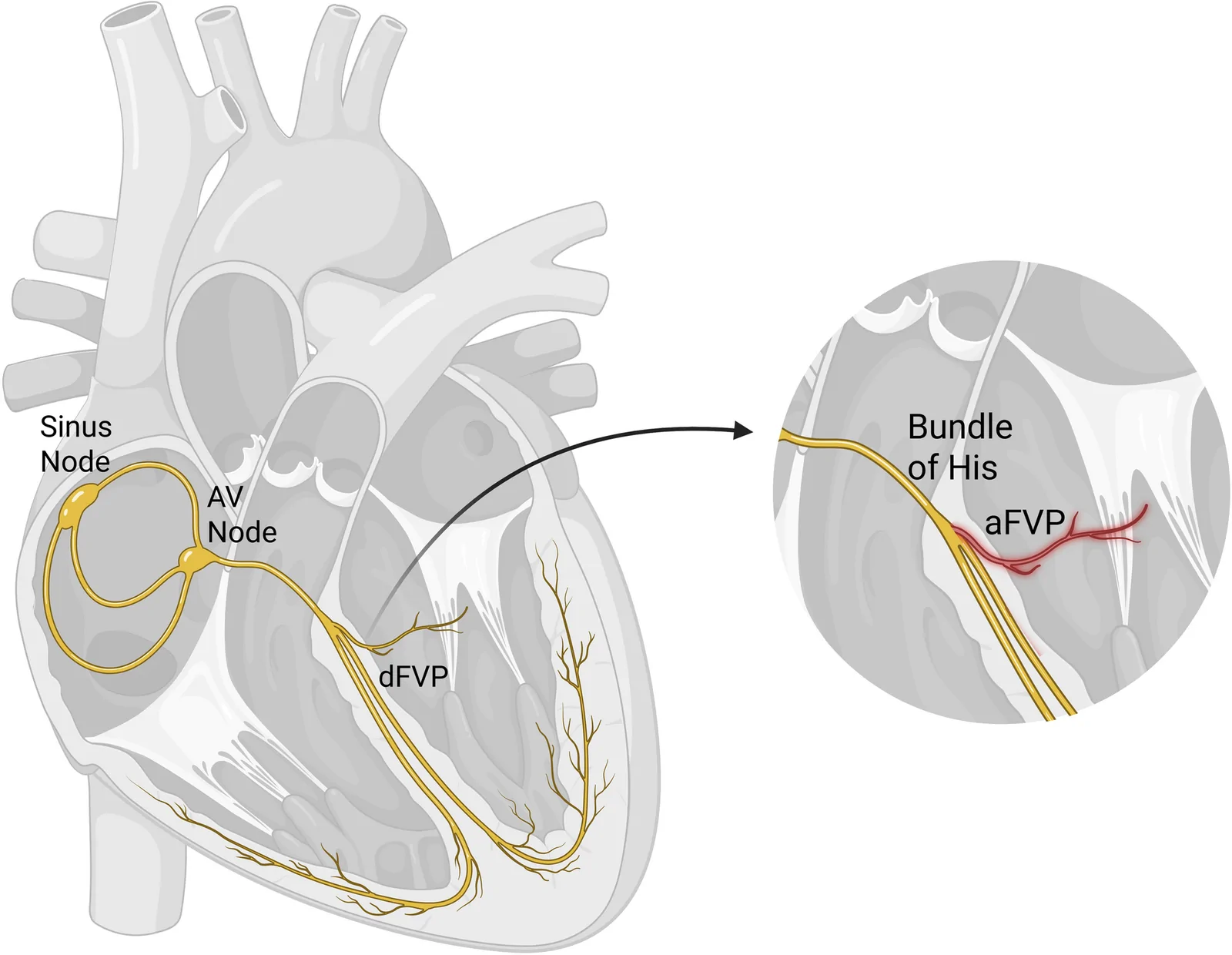
Schematic representation of the human heart conduction system highlighting fasciculoventricular pathways (FVPs). Illustration of a dormant FVP (dFVP) originating from the bundle of His and inserting directly into the ventricular myocardium. Zoomed inset: Activated FVP (aFVP), driven by glycogen accumulation and metabolic altered signaling, coursing from the bundle of His into the ventricular myocardium representing the preexcitation phenotype in PRKAG2 cardiomyopathy. The normal atrioventricular conduction axis [sinoatrial node $\:\to\:$ AV node $\:\to\:$ bundle of His $\:\to\:$ bundle branches $\:\to\:$ ventricles] is shown for comparison

These findings challenge the previously proposed hypothesis of structural disruption of the annulus fibrosus due to glycogen accumulation in cardiomyocytes, a hypothesis presumably consistent with the histological findings in Asn488Ile transgenic mice [59]. Moreover, the evidences suggest that glycogen accumulation in muscle fibers leads to VPE by making ubiquitous dormant fasciculoventricular pathways electrically active (Fig. 1).

These electrophysiological alterations underlie two primary mechanisms of SCD in PCS. The first is an arrhythmogenic pathway: early-onset atrial tachyarrhythmias (such as atrial flutter) conducted rapidly over an activated FVP bypass normal AV nodal delay, triggering rapid ventricular rates that can degenerate into fatal ventricular fibrillation [12, 55] The second is a degenerative conduction pathway: progressive glycogen accumulation directly damages specialized conduction tissue in the SAN and AV node, leading to sudden complete AV block, severe bradycardia, and asystole [11, 44 ].

### Clinical Management and Future Perspectives

To date, no disease-specific therapeutic protocols for PCS have been validated in randomized clinical trials. Patient care aligns with broader guidelines for hypertrophic cardiomyopathy, such as the 2024 AHA/ACC guidelines, with the essential caveat that PCS is a metabolic storage disease distinct from sarcomeric HCM [60]. Current clinical management remains supportive. It focuses on arrhythmia surveillance, antiarrhythmic pharmacotherapy, catheter ablation of accessory pathways, PPM implantation for conduction disease, and standard heart failure management for systolic dysfunction [9]. PPM are indicated in cases of advanced AV block or severe symptomatic bradycardia. Meanwhile, implantable cardioverter-defibrillators (ICDs) are reserved for high-risk patients to prevent SCD. Risk stratification for ICD candidacy relies on specific markers, including a family history of premature SCD, syncope of suspected arrhythmic etiology, extreme hypertrophy, non-sustained ventricular tachycardia, and adverse cardiovascular MRI tissue characterization patterns [3]. Electrophysiological study and catheter ablation are indicated for accessory pathways to prevent rapid ventricular response or degenerating atrial arrhythmias [3, 60]. Management of heart failure with reduced ejection fraction follows standard guideline-directed medical therapy (GDMT), including RAAS inhibitors, SGLT2 inhibitors, and mineralocorticoid receptor antagonists (MRAs). Beta-blockers must be titrated with extreme caution prior to PPM placement. Cardiac transplantation remains the definitive option for end-stage disease refractory to medical management [3, 61].

**Fig. 2:**
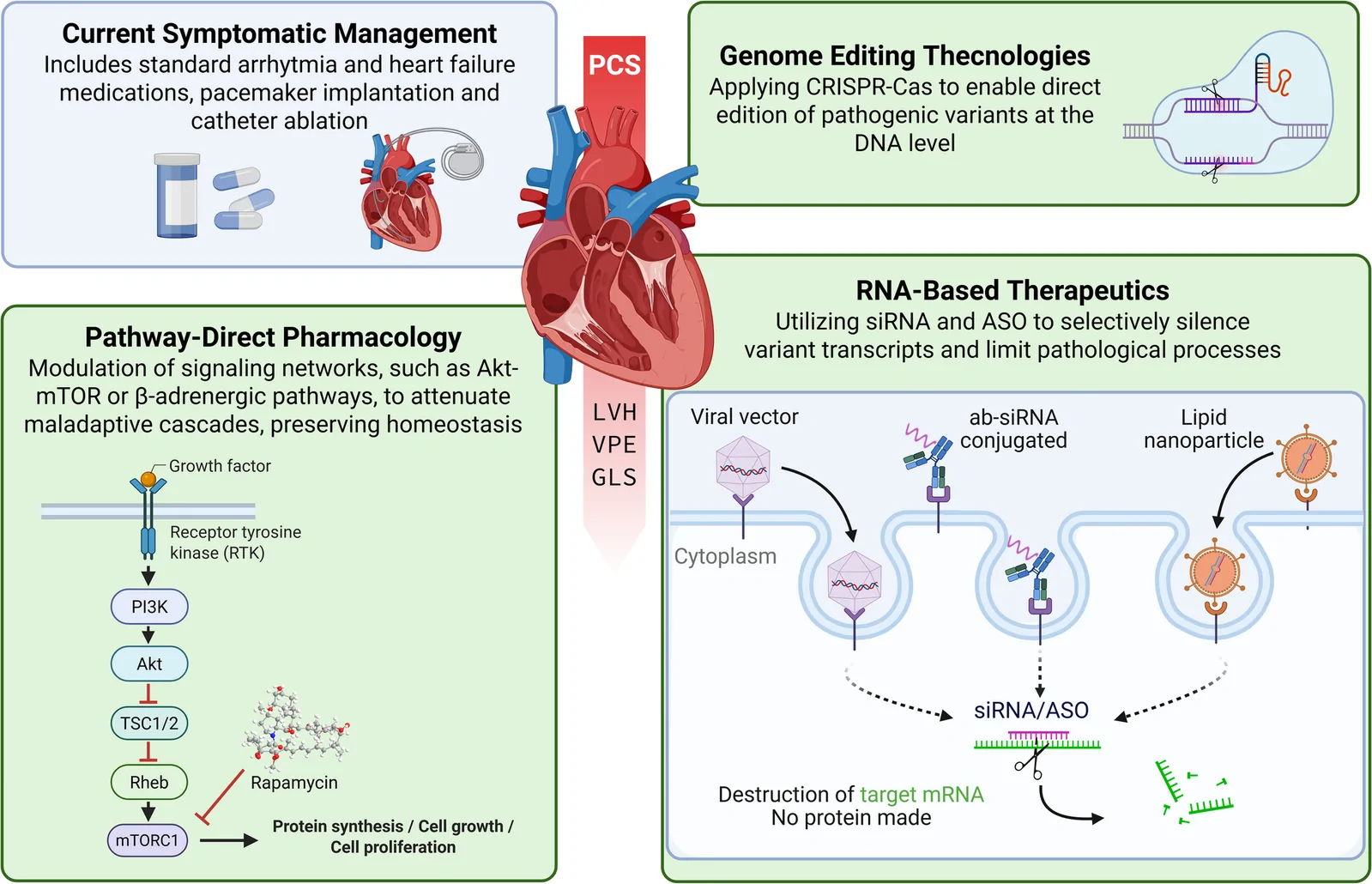
Therapeutic paradigm transition in PRKAG2 cardiac syndrome. Summary of the transition from supportive management (rhythm monitoring, pacemaker/ICD implantation, catheter ablation, standard heart failure therapy) to targeted precision medicine strategies. Mechanisms aimed at addressing primary disease drivers include RNA-based gene silencing (siRNA, ASO), pathway-directed pharmacology (mTOR/Akt inhibitors, beta-blockers), and CRISPR-based gene editing delivered via cardiotropic vectors or lipid nanoparticles. LVH, left ventricular hypertrophy; VPE, ventricular preexcitation; GLS, glycogen storage

Future therapeutic paradigms for PCS are progressively moving beyond conventional symptomatic management toward mechanism-based and potentially disease-modifying strategies (Fig. 2). Alglucosidase alfa [recombinant human alpha-glucosidase (rhGAA)] is the standard enzyme replacement therapy (ERT) for Pompe disease (a lysosomal glycogen storage disease), but clinical evidence suggests potential cross-over efficacy for cytoplasmic glycogenoses like PCS. In a key clinical case, a pediatric patient carrying the *PRKAG2* Gly100Ser mutation was initially misdiagnosed with Pompe disease and started on rhGAA. The treatment produced significant improvements in muscle strength, motor function, and seizure control. When the patient’s diagnosis was corrected and ERT was discontinued for 14 months, he suffered a severe clinical and motor decline. Reinitiating rhGAA successfully restored his strength and motor gains [62].

As mentioned early, Akt/mTOR pathway is abnormally upregulated in PCS. The Lys475Glu variant triggers an Akt-independent, hyperactive mTOR signaling cascade characterized by the marked hyperphosphorylation of p70S6K and 4E-BP1, which directly promotes cell enlargement. While short-term treatment is insufficient to reverse this genetic hypertrophic phenotype, sustained pharmacological inhibition with rapamycin (50 nM) over 3.5 to 5 days successfully rescues the cell hypertrophy in mutant H9c2 cardiomyocytes [63]. Moreover, beta-blockers like metoprolol have been shown to attenuate Akt/mTOR hyperactivation in Arg302Gln models, blunting hypertrophic signaling [6].

Since many prevalent mutations cause a baseline gain-of-function in AMPK activity that drives glycogen deposition, directly inhibiting AMPK offers a straightforward therapeutic approach. Differentiated human induced pluripotent stem cell-derived cardiomyocytes (iPSC-CMs) carrying the Arg302Gln mutation exhibit increased basal AMPK activity, massive glycogen vacuoles, and significant cellular enlargement. Pharmacological inhibition of AMPK with the small molecule Compound C (1–2 µM) for 7 days successfully cleared abnormal glycogen accumulations and downregulated key hypertrophic and sarcomeric gene markers in patient-specific iPSC-CMs [64].

RNA-based therapies have emerged as a particularly promising frontier. Small interfering RNAs (siRNAs) and antisense oligonucleotides (ASOs) can be engineered to selectively silence mutant *PRKAG2* transcripts, suppressing abnormal AMPK signaling and reducing myocardial glycogen storage. Allele-specific silencing is particularly attractive, as it targets the mutant allele while leaving the wild-type allele intact. One of the most clinically advanced precision therapies for *PRKAG2* is an antibody-oligonucleotide conjugate designed for selective cardiac delivery. An antibody-oligonucleotide conjugate (AOC 1072/ATR 1072; Avidity Biosciences, now Atrium Therapeutics) targets the transferrin receptor-1 to deliver siRNA directly and selectively to cardiac muscle tissue. In non-human primates, this therapy achieved approximately 85% knockdown of *PRKAG2* mRNA, and it successfully reduced myocardial glycogen accumulation in a murine model of PCS without inducing adverse electrophysiological or ECG effects [65].

Furthermore, gene-editing platforms—including CRISPR-Cas9, base editing, and prime editing—offer the potential for permanent correction of pathogenic *PRKAG2* variants at the genomic level. In His530Arg mutant mouse models, researchers successfully combined adeno-associated virus-9 (AAV9) with CRISPR-Cas9 to selectively cleave and disrupt the dominant disease-causing mutated allele while leaving the healthy wild-type allele fully functional. A single systemic injection of AAV9-Cas9/sgRNA at postnatal stages reversed cardiac hypertrophy, cleared glycogen-filled vacuoles, improved systolic function (ejection fraction/fractional shortening), and reduced pre-excitation incidence. Crucially, low editing efficiencies (~ 2.6% to 6.5%) were sufficient to achieve dramatic therapeutic rescue [66].

While promising, translational barriers including off-target effects, cardiac delivery efficiency, immunogenicity, and long-term genomic stability must be rigorously addressed before clinical application.

## Conclusion

PCS represents a distinct metabolic storage etiology within the broader spectrum of hypertrophic cardiomyopathies. Comprehensive clinical characterization and genotype-phenotype mapping have established crucial diagnostic red flags that are essential for differentiating PCS from classical sarcomeric hypertrophy. At the cellular level, unraveling the molecular architecture of PCS has revealed a multi-tiered pathophysiological mechanism. Pathogenic variants induce chronic AMPK overactivation, which drives intracellular glycogen storage through G6P-mediated allosteric activation of glycogen synthase. This process follows a biphasic temporal model, evolving from early GLUT4-mediated glucose flooding to late feedback down-regulation of metabolic flux. Furthermore, myocardial hypertrophy is actively driven by hyperactivation of the Akt–mTOR signaling axis, while VPE is predominantly mediated by the unmasking and electrical activation of dormant fasciculoventricular pathways. Although contemporary clinical management remains largely supportive — focusing on arrhythmia surveillance, early PPM or ICD implantation, and standard heart failure therapy —the management paradigm for PCS is rapidly advancing. Emerging precision medicine frontiers, including allele-specific RNA silencing, pathway-targeted pharmacological inhibitors, and CRISPR-Cas9 gene editing, offer genuine potential to transition PCS care from symptomatic management to targeted, disease-modifying therapies. Overcoming key translational barriers regarding cardiac delivery efficiency and long-term safety will be decisive in bringing these personalized gene and molecular strategies to clinical practice. 

## Supplementary Information

Supplementary Material 1.

## Data Availability

Data sharing is not applicable to this article as no new datasets were generated or analyzed during the current study.

## Abbreviations

4EBP1: Eukaryotic translation initiation factor 4E-binding protein 1
AF: Atrial fibrillation
(a/d)FVP: (activated/dormant) Fasciculoventricular pathway
Akt: Protein kinase B
AMPK: Adenosine monophosphate-activated protein kinase
AOC: Antibody-oligonucleotide conjugate
ASO: Antisense oligonucleotide
ATP: Adenosine triphosphate
AV: Atrioventricular
AAV9: Adeno-associated virus-9
CBM: Carbohydrate-binding module
CBS: Cystathionine$\:\beta\:$-synthase
CRISPR-Cas9: Clustered Regularly Interspaced Short Palindromic Repeats-associated protein 9
DCM: Dilated Cardiomyopathy
DNA: Deoxyribonucleic acid
ECG: Electrocardiography
ERT: Enzyme replacement therapy
FDG: Fluorodeoxyglucose ($$\:{}_{}{}^{18}\mathrm{F}$$)
FoXO: Forkhead box O
G6P: Glucose-6-phosphate
GLUT4: Glucose transporter type 4
GS: Glycogen synthase
HCM: Hypertrophic cardiomyopathy
HCN4: Hyperpolarization-activated cyclic nucleotide-gated potassium channel 4
ICD: Implantable cardioverter-defibrillator
$$\:{I}_{f}$$: Funny current
iPSC: Induced pluripotent stem cell
LGE: Late gadolinium enhancement
LVH: Left ventricular hypertrophy
MRI: Magnetic Resonance Imaging
mTOR: Mechanistic target of rapamycin
mTORC1: Mechanistic target of rapamycin complex 1
p70S6K: p70 ribosomal S6 kinase
PCS: PRKAG2 cardiac syndrome
PET: Positron emission tomography
PFK1 / PFK2: Phosphofructokinase 1 / 2
PPM: Permanent pacemaker
RNA: Ribonucleic acid
SAN: Sinoatrial node
SCD: Sudden cardiac death
siRNA: Small interfering RNA
VPE: Ventricular preexcitation
WPW: Wolff-Parkinson-White

## Declarations

### Literature Search Strategy

Literature searches were conducted across PubMed and Google Scholar for relevant studies published between January 2000 and May 2026. Search terms were structured around the key thematic sections of this review. Search results were screened by title and abstract, and peer-reviewed articles directly addressing the narrative scope in indexed journals were selected for inclusion.
