# Supplementary material for: PRKAG2 Cardiac Syndrome: A Comprehensive Review of Clinical Manifestations, Pathophysiological Mechanisms, and Precision Medicine Frontiers
Source: J Cardiovasc Transl Res. 2026 Nov 5;19(1):142. doi: 10.1007/s12265-026-10851-y (PMC13739962; doi:10.1007/s12265-026-10851-y)
Supplement: Supplementary file 1 [file 12265_2026_10851_MOESM1_ESM.docx]

Conflict of Interest: The authors declare that they have no conflict of interest.

Glauber Monteiro Dias, on behalf of all co-authors

*Laboratório de Biologia Celular e Tecidual
Universidade Estadual do Norte Fluminense Darcy Ribeiro - UENF
Avenida Alberto Lamego, 2000. Parque Califórnia
Campos dos Goytacazes – RJ. Brazil*

*Postal Code: 28013-602
Tel: +55 21 998070324*

*glauber@uenf.br*
